# Understanding Natural Cognition in Everyday Settings: 3 Pressing Challenges

**DOI:** 10.3389/fnhum.2018.00386

**Published:** 2018-09-27

**Authors:** Francisco J. Parada

**Affiliations:** Laboratorio de Neurociencia Cognitiva y Social, Facultad de Psicología, Universidad Diego Portales, Santiago, Chile

**Keywords:** mobile brain/body imaging (MoBI), 4E-cognition, methodologic considerations, epistemology, natural cognition

On July 2018, 156 people gathered together in Berlin to share and discuss work and ideas flourishing from one of the most exciting concepts about the mind: natural cognition (Makeig et al., 2009; Gramann et al., 2014). These researchers are looking forward to move away from *good old-fashioned cognitive science* (GOFACS) and transition to the next phase in the study of the biophysics of human experience. Thus, the 3rd International Mobile Brain/Body Imaging (MoBI) Conference was carried out at TU Berlin. Given each work's complexity, it would be futile to try to distinguish *which* or *how many* disciplines were represented during the conference. Talks ranged from novel software and hardware to novel experiments and analyses to clinical and artistic interventions. Thus evidencing the truly multidisciplinary scientific effort carried by the MoBI community: attendants' background ranged from physics, engineering, and neuroscience to psychology, arts and therapeutics. Furthermore, Scott Makeig's closing keynote talk provided an inspiring account on the development, current state, and future challenges of MoBI, from the point of view of the very man who has spearheaded some of the most relevant aspects of the movement (Makeig et al., 2009; Gramann et al., 2010, 2011, 2014; Gwin et al., 2011; Ojeda et al., 2014; Liao et al., 2015). The MoBI community is building a research program for the 21st century allowing the understanding of natural cognition in everyday settings. Notwithstanding relevant advancements reached during the first 10 years of its existence (Gramann et al., 2014; Ladouce et al., 2017), there still are important pitfalls to keep in mind as the field moves forward. Therefore, I would like to present the reader with what appears to me -after experiencing the meeting- three pressing short-term challenges faced by the MoBI community.

## Challenge N° 1: systematically extending our laboratories

**Figure 1:**
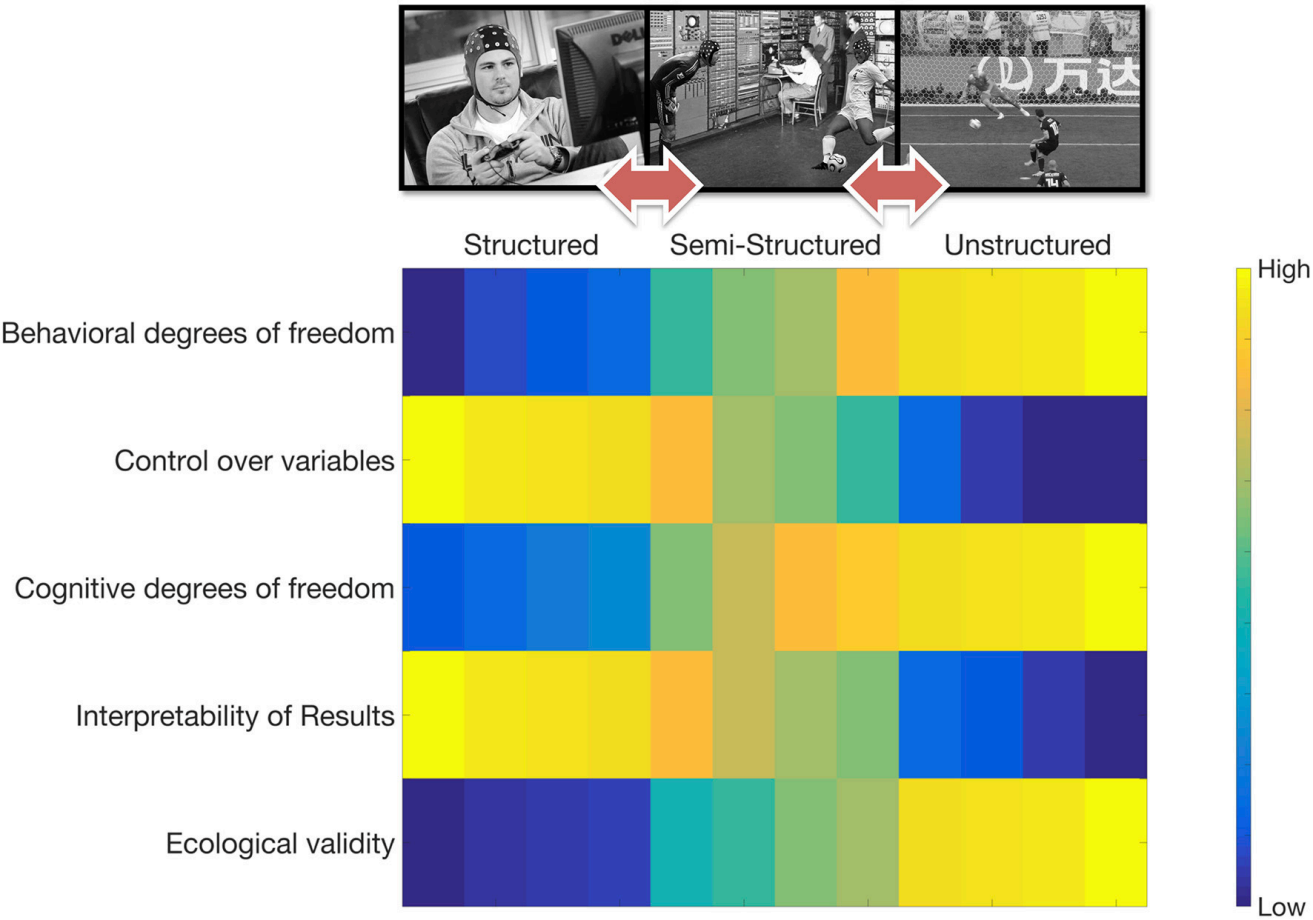
The continuous between structured experimental designs and unstructured ones, the impact of design decisions on some dimensions such as ecological validity and cognitive degrees of freedom are depicted. MoBI experiments could be carefully designed in order to be escalable, allowing (i) the exploration of brain/body dynamics emerging from structured and controlled conditions on robust findings [e.g., N2/P3 complex (Folstein and Van Petten, 2008)], (ii) testing previous results in semi-structured complex yet fairly controlled situations [e.g. (Malcolm et al., 2017)], (iii) pushing new hypothesis into complex unstructured settings, and (iv) returning back to a more structured settings if needed. Structured experimental designs are characterized by increasing internal validity at the cost of ecological validity. Left column shows a typical structured experimental setting, where a micro-version of a complex task can be implemented. Such a structured task could range from basic attentional deployment paradigm to a fully-fledged goal-kicking/catching simulator. Furthermore, this experiment could be implemented in both one-person or hyperscanning setups. Multi-modal data acquisition is very possible and highly encouraged in structured settings. Semi-structured experimental designs are characterized by providing some control over experimental variables and more behavioral/cognitive degrees of freedom through allowing more real-life behaviors at reduced ecological validity. Middle column depicts a semi-structured interactional setting where some aspects of natural cognition are preserved. As in real-life, the behavioral goal will be to score (in the case of the kicker) and to catch the ball (in the case of the goalkeeper). Even though multi-modal data acquisition still is a complex task, given the semi-structured nature of the design (e.g., trials, experimenter-controlled timings, etc.), data analysis and interpretation might be relatively straightforward. Finally, unstructured experimental designs are (almost) real-life situations where brain/body datasets are collected. In these setups macro-cognitive states unfold where participants have large behavioral/cognitive degrees of freedom at the possible cost of internal validity and interpretability of results. Right column depicts a natural unstructured interactional setting where collective time-pressured engaged decisions are made, in this case the common goal is to score. Considering these three levels as a natural continuum provides grounds for advancing the field. EEG depictions courtesy of ANT Neuro (The Netherlands).

MoBI -by definition- implies extending our structured and controlled laboratory settings toward semi- or unstructured ones allowing cognitive acts to unfold naturally in all its complexity. However, there still is very little consensus on what would constitute the “standard MoBI experiment,” if there is such a thing. For some researchers the answer might lie in controlling—as carefully as possible—the type of interaction on which participants engage. Generating thus well-defined and identifiable experimental conditions *embedded* in more ecological settings than the laboratory (e.g., Malcolm et al., 2017; Pizzamiglio et al., 2017). These type of experiments are—to me—the epitome of laboratory extensions to the real world through translation of “classic” structured settings (and therefore “classic” effects) to the MoBI paradigm, hopefully leading toward new hypotheses—unreachable within the lab. In contrast, a more intuitive approach is generating open interactional setups, allowing for a virtually intractable number of degrees of freedom for each participant's cognitive acts (e.g., Herrera-Arcos et al., 2017). While it could be thought that unstructured settings might be the conceptual holy grail of the MoBI paradigm, it is important to clarify that semi-structured settings try to maintain the possibility of identifying experimental conditions, whilst no *a priori* patterns can be determined from unstructured settings. In contrast, benefits reached in terms of ecological validity are lost when decreasing degrees of freedom (Figure 1). Currently, no consensus exists on which approach should be taken and, in the long run, I think that it does not matter if consensus is ever reached. The real pitfall here is thinking that a specific position in the continuous between structured and unstructured settings is more important than others, leading to disqualifying, abandoning or ignoring progress at other points of the spectrum. These are complementary approaches that should be run in parallel as much as possible (Ladouce et al., 2017).

Personally, I would like to see *escalable experimental designs* (EED) becoming the standard in MoBI research, leaving completely unstructured setups as a goal for longer-term research programs or as relevant proof-of-concept for hardware/software/intervention development. EEDs are characterized by carefully designing highly structured experiments always having in mind the possibility to be later extended—on a step—wise fashion toward the real world- ultimately becoming unstructured, dynamic, and social real-world settings with solid data-driven grounds for hypothesis testing. EEDs are an epistemological stance, where reductionism is no longer a concern as the ultimate goal is laying grounds for *novel* hypotheses to be tested in the wild. Another relevant aspect of EEDs is that they can be thought of as emergents of a naturally distributed research program; systematically extending the laboratory toward the real world as well as connecting different research groups working the same designs at different points in the spectrum.

## Challenge N°2: neurocentrism 2.0

In Stefan Debener's presentation, a clear goal was presented: developing *Transparent MoBI* (Debener et al., 2015; Bleichner and Debener, 2017; Debener, 2018). This is bio-behavioral measurements that do not obstruct—in any way—the agent's natural behavior. Debener's dream system would measure (neuro)physiological dynamics along with eye movements, body topology, spatial location, etc., without interfering in the agent's evolutionarily-given degrees of freedom for interaction. Unfortunately, this dream system is not yet available (Debener et al., 2015; Mihajlovic et al., 2015; Goverdovsky et al., 2016; Bleichner and Debener, 2017; Ladouce et al., 2017; Debener, 2018). However, obtrusive/bulky or not, current systems allow us to actually measure several of these signals. Within this context -although significant improvement from GOFACS- the fact that some of our work might only be interested in measuring and reporting *brain dynamics in the wild* might be misleading for the extended scientific community in the long run. Closing the gap between acquired mobile and laboratory signal quality is crucial at this stage of development (Mihajlovic et al., 2015; Bleichner and Debener, 2017). Thus focusing *only* on brain signals is highly appropriate within this context. However, acknowledging this focus as a methodological need and not as an epistemological decision *does* make a difference.

On the one hand, the MoBI paradigm rather explicitly positions the brain/body-in-the-world system as the new epistemological object for cognitive science (Parada and Rossi, 2018). On the other hand, *neurocentrism* has been so radically implanted in our mindsets (Clark, 1997, 2008) that we must consciously make efforts so it does not make its way into the MoBI community as well. In the current brain-dominated cultural landscape (Satel and Lilienfeld, 2013), we might—unknowingly—be creating a neurocentric standard for current and future MoBI experiments. Such a standard might be as difficult to deconstruct as the event-related potential (ERP) paradigm once was, where people ended up “acquiring ERP” directly from the brain. As if ERP waves were natural objects to be collected, not the mathematical outcome of lack of computers in the late 30's (Davis et al., 1939) and low computing power in the 60's (Galambos and Sheatz, 1962). Given neuroscientists' historic bias toward neurocentrism (Clark, 1997, 2008), we must be weary to not carry out this bias any further.

Personally, I think epistemologically defining cognition not only as *Embodied* but also as *Embedded* in its socio-cultural context, as well as *Extended* and *Enacted* onto the physical and social world is key (Parada and Rossi, 2018). Moreover, this *4E-cognition* (Menary, 2010) approach should be accompanied with proper *4E* data acquisition. Understanding natural cognition in everyday settings will inevitably induce different states in the brain/body system (Bronfenbrenner, 1977; Ladouce et al., 2017). These new states will arise from developmental, demographic, social, and life-style factors; ontogeny. Most of these data sources are usually considered as not relevant for GOFACS as we usually not even register them in our data acquisition protocols (Varela, 1996). Sometimes we even try to “control” some of these variables by providing our participants with some life-style-disrupting instructions such as “don't drink coffee before the experiment.” Some other times, large longitudinal (neuro)physiological datasets might be acquired from specific subsets of general population while only collecting basic demographic information (age, sex, etc.). I personally envision an integration between brain signals and other data sources from both micro- and macro-levels of the brain/body system, longitudinally acquired. This is, other physiological patterns (such as electromyography, electrogastrography, electrocardiography, hormonal profiles, among others; e.g., Brouwer et al., 2018 in this issue) along with demographical, lifestyle, and social information such as eating (McGarel et al., 2014; Ojha et al., 2015; Zuker, 2015) and sleeping habits (Cedernaes et al., 2015; Dashti et al., 2015; Irwin, 2015), current employment situation/satisfaction (Faragher et al., 2013; Benach et al., 2014), drug/substance usage (Volkow et al., 2016) to name a few (Mattson, 2015). After all, for better or worse MoBI is a “big data” project already. A good reason to fully accept this, can be found on how only focusing and testing isolated predictors led to a historic near-chance success rates of suicide prediction (Franklin et al., 2017). Importantly, combining hundreds of predictors leads to >80% accuracy using the predictive/prospective analytics framework including machine learning (Walsh et al., 2017). The correct implementation of such an integrated approach implies rejection of neurocentrism. Further, this is an opportunity for the unification of sociology, anthropology, and other non-brain biased social and health scientists into the MoBI paradigm.

## Challenge N°3: data management, from storage to reporting

Analyzing and interpreting MoBI datasets might be as complex of a process as collecting them. As a community we should emphasize, promote, and encourage replication studies, re-analises of already published MoBI effects (e.g., adding a “replications” section at the conference), as well as collaborative data mining of existing datasets (e.g., adding a “hackaton” session at the conference). Furthermore, dataset sharing is excellent practice and will only make the MoBI community stronger (e.g., Brantley et al., 2018). As datasets increase in number and complexity, non-trivial practical challenges arise for an open-science MoBI: from simply saving data in a hard drive to how analyze, report, and interpret it.

First, current containerization initiatives (for details see Gorgolewski et al., 2016) have been developed outside the multimodal framework where MoBI operates. The fact that we might be able to still retrace most existing MoBI datasets, should motivate the community to make active efforts for standardization of containerization practices as a relevant step for facilitating data sharing and advancing the field forward.

Second, even if we could contain our data in similar formats, sharing these data will not be straightforward. There are legal issues that might directly obstruct and interfere with the open science framework. For example, in Chile there are no specific laws about research data management. Therefore the closest law must be applied, which is about patient medical records. This law declares the service provider as responsible for keeping the records confidential for at least 15 years and forbids unauthorized third-party usage of those records. In the USA, the NIH has recently redefined the meaning of “clinical trials” (Hudson et al., 2016) whilst the General Data Protection Regulation in the European Union started its regime in May 2018, with yet unknown impacts to data sharing.

Finally, when properly organized and legally distributed data is at hand we encounter analysis, interpretation and reporting decisions. Enough evidence has already been gathered about the dangers of small sample-based research and its correspondent *p*-value-based conclusions (Yarkoni, 2009). Large-sample studies might not be the norm in the near future for MoBI. Therefore, integrating some safe-net practices into the very core of MoBI studies might be a good idea. Such practices include: (i) refrain drawing conclusions from fixed *p*-value thresholds (i.e., frequentist fallacies, see Wagenmakers, 2007; Nieuwenhuis et al., 2011), (ii) avoiding scarcity of illustrations depicting data and results (usually biased only toward graphing positive results!). More and more journals, such as *Frontiers*, are offering a very large number of figures per publication, let's use them, and (iii) including confidence intervals in illustrations and result reports. Excellent materials on how to avoid these and other common data analysis pitfalls have been recently developed, for example see (Pernet et al., 2011, 2013; Ehinger and Dimigen, 2018; Wilcox and Rousselet, 2018) to highlight a few.

## Concluding remarks

The last decade has seen the emergence of the MoBI paradigm and, after attending the 3rd International MoBI conference, we can be confident that the community stands strong toward its second decade. However, given that the field is still fledgling, here I have highlighted some pressing pitfalls. Avoiding those will mean a focus on maximizing replication both at the individual and group levels. This goal can be reached through the development of EEDs as well as implementing good practices (e.g., data sharing, specifying, and sharing parameters, exact procedures and instrumentation, etc.). Furthermore, taking into account that the brain/body system is embedded in and extended toward the world (Parada and Rossi, 2018), data acquisition *will* be more complex than we can imagine. When high-quality *Transparent EEG* is accomplished (Debener et al., 2015; Mihajlovic et al., 2015; Goverdovsky et al., 2016; Bleichner and Debener, 2017; Debener, 2018), (neuro)physiological recordings will be trivial and other data sources will be included as part of the standard MoBI setup. These data will truly help us model the unique as well as shared aspects of cognition and the biophysics of human experience.

## Author contributions

The author confirms being the sole contributor of this work and has approved it for publication.

### Conflict of interest statement

The author declares that the research was conducted in the absence of any commercial or financial relationships that could be construed as a potential conflict of interest.

## References

[B1] Benach J. Vives A. Amable M. Vanroelen C. Tarafa G. Muntaner C. (2014). Precarious employment: understanding an emerging social determinant of health. Ann. Rev. Public Health 35, 229–253. 10.1146/annurev-publhealth-032013-182500 24641559

[B2] Bleichner M. G. Debener S. (2017). Concealed, unobtrusive ear-centered EEG acquisition: cEEGrids for transparent EEG. Front. Hum. Neurosci. 11:163 10.3389/fnhum.2017.00163 28439233 PMC5383730

[B3] Brantley J. A. Luu T. Nakagome S. Zhu F. Contreras-Vidal J. (2018). Full body mobile brain-body imaging data during unconstrained locomotion on stairs, ramps, and level ground. Sci Data 5:180133. 10.1038/sdata.2018.133 29989591 PMC6038848

[B4] Bronfenbrenner U. (1977). Toward an experimental ecology of human devel- opment. Am. Psychol. 32:513 10.1037/0003-066X.32.7.513

[B5] Brouwer A. M. van Dam E. van Erp J. B. F. Spangler D. P. Brooks J. R. (2018). Improving real-life estimates of emotion based on heart rate: a perspective on taking metabolic heart rate into account. Front. Hum. Neurosci. 12:284 10.3389/fnhum.2018.00284 30061818 PMC6054929

[B6] Cedernaes J. Schi oth H. B. Benedict C. (2015). Determinants of short- ened, disrupted, and mistimed sleep and associated metabolic health con- sequences in healthy humans. Diabetes. 64, 1073–1080. 10.2337/db14-1475 25805757

[B7] Clark A. (1997). Being There. Cambridge, MA: MIT Press.

[B8] Clark A. (2008). Supersizing the Mind: Embodiment, Action, and Cognitive Extension. Oxford: Oxford University Press.

[B9] Dashti H. S. Scheer F. A. Jacques P. F. Lamon-Fava S. Ordov as J. M. (2015). Short sleep duration and dietary intake: epidemiologic evidence, mechanisms, and health implications. Adv. Nutr. 6, 648–659. 10.3945/an.115.008623 26567190 PMC4642416

[B10] Davis H. Davis P. A. Loomis A. L. Harvey E. N. Hobart G. (1939). Electrical reactions of the human brain to auditory stimulation during sleep. J. Neurophysiol. 2, 500–514. 10.1152/jn.1939.2.6.500

[B11] Debener S. (2018). Developing portable, mobile, and transparent EEG, in Proceedings of the 3rd International Mobile Brain/Body Imaging Conference. eds. Gramann Berlin K. 5.

[B12] Debener S. Emkes R. De Vos M. Bleichner M. (2015). Unobtrusive am- bulatory EEG using a smartphone and flexible printed electrodes around the ear. Sci. Rep. 5:16743 10.1038/srep16743 26572314 PMC4648079

[B13] Ehinger B. V. Dimigen O. (2018). Unfold: An integrated toolbox for overlap correction, non-linear modeling, and regression-based EEG analysis. bioRxiv, 360156. 10.1101/360156 PMC6815663 31660265

[B14] Faragher E. B. Cass M Cooper C. L. (2013). The relationship between job satisfaction and health: a meta-analysis, in From Stress to Wellbeing Volume 1, ed Cooper C. L. (London: Palgrave Macmillan). 10.1057/9781137310651_12 PMC1740950 15657192

[B15] Folstein J. R. Van Petten C. (2008). Influence of cognitive control and mismatch on the N2 component of the ERP: a review. Psychophysiology 45, 152–170. 10.1111/j.1469-8986.2007.00602.x 17850238 PMC2365910

[B16] Franklin J. C. Ribeiro J. D. Fox K. R. Bentley K. H. Kleiman E. M. Huang X. (2017). Risk factors for suicidal thoughts and behaviors: a meta-analysis of 50 years of research. Psychol. Bull. 143:187 10.1037/bul0000084 27841450

[B17] Galambos R. Sheatz G. C. (1962). An electroencephalograph study of classical conditioning. Am. J. Physiol. Cont. 203, 173–184. 10.1152/ajplegacy.1962.203.1.173 13896298

[B18] Gorgolewski K. J. Auer T. Calhoun V. D. Craddock R. C. Das S. Duff E. P.. (2016). The brain imaging data structure, a format for organizing and describing outputs of neuroimaging experiments. Sci. Data 3:160044. 10.1038/sdata.2016.44 27326542 PMC4978148

[B19] Goverdovsky V. Looney D. Kidmose P. Mandic D. P. (2016). In-Ear EEG from viscoelastic generic earpieces: robust and unob- trusive 24/7 monitoring. IEEE Sens J. 16, 271–277. 10.1109/JSEN.2015.2471183

[B20] Gramann K. Ferris D. P. Gwin J. Makeig S. (2014). Imaging natural cognition in action. Int. J. Psychophysiol. 91, 22–29. 10.1016/j.ijpsycho.2013.09.003 24076470 PMC3983402

[B21] Gramann K. Gwin J. T. Bigdely-Shamlo N. Ferris D. P. Makeig S. (2010). Visual evoked responses during standing and walking. Front. Hum. Neurosci. 4:202. 10.3389/fnhum.2010.00202 21267424 PMC3024562

[B22] Gramann K. Gwin J. T. Ferris D. P. Oie K. Jung T. P. Lin C. T.. (2011). Cognition in action: imaging brain/body dynamics in mobile humans. Rev. Neurosci. 22, 593–608. 10.1515/RNS.2011.047 22070621

[B23] Gwin J. T. Gramann K. Makeig S. Ferris D. (2011). Electrocortical activity is coupled to gait cycle phase during treadmill walking. Neuroimage 54, 1289–1296. 10.1016/j.neuroimage.2010.08.066 20832484

[B24] Herrera-Arcos G. Tamez-Duque J. Acosta-De-Anda E. Kwan-Loo K. de Alba M. Tamez-Duque U. (2017). Modulation of neural activity during guided viewing of visual art. Front. Hum. Neurosci. 11:581 10.3389/fnhum.2017.00581 29249949 PMC5714858

[B25] Hudson K. L. Lauer M. S. Collins F. S. (2016). Toward a new era of trust and transparency in clinical trials. JAMA 316:1353 10.1001/jama.2016.14668 27636028 PMC5101947

[B26] Irwin M. R. (2015). Why sleep is important for health: a psychoneuroimmunology perspective. Ann. Rev. Psychol. 66, 143–172. 10.1146/annurev-psych-010213-115205 25061767 PMC4961463

[B27] Ladouce S. Donaldson D. I. Dudchenko P. A. Ietswaart M. (2017). Understanding minds in real-world environments: toward a mobile cognition approach. Front. Hum. Neurosci. 10:694 10.3389/fnhum.2016.00694 28127283 PMC5226959

[B28] Liao Y. Acar Z. A. Makeig S. Deak G. (2015). EEG imaging of toddlers during dyadic turn-taking: Mu-rhythm modulation while producing or observing social actions. NeuroImage 112, 52–60. 10.1016/j.neuroimage.2015.02.055 25731992

[B29] Makeig S. Gramann K. Jung T.-P. Sejnowski T. J. Poizner H. (2009). Linking brain, mind and behavior. Int. J. Psychophysiol. 73, 95–100. 10.1016/j.ijpsycho.2008.11.008 19414039 PMC2796545

[B30] Malcolm B. R. Foxe J. J. Butler J. S. Mowrey W. B. Molholm S. De Sanctis P. (2017). Long-term test-retest reliability of event-related potential (ERP) recordings during treadmill walking using the mobile brain/body imaging (MoBI) approach. Brain Res. 10.1016/j.brainres.2017.05.021 [Epub ahead of print]. 28532853 PMC7209996

[B31] Mattson M. P. (2015). Lifelong brain health is a lifelong challenge: from evolutionary principles to empirical evidence. Age. Res. Rev. 20, 37–45. 10.1016/j.arr.2014.12.011 25576651 PMC4346441

[B32] McGarel C. Pentieva K. Strain J. J. McNulty H. (2014). Emerging roles for folate and related B-vitamins in brain health across the life- cycle. Proc. Nutr. Soc. 74, 46–55. 10.1017/s0029665114001554 25371067

[B33] Menary R. (2010). Introduction to the special issue on 4E cognition. Phenomenol. Cogn. Sci. 9, 459–463. 10.1007/s11097-010-9187-6

[B34] Mihajlovic V. Grundlehner B. Vullers R. Penders J. (2015). Wear- able wireless EEG solutions in daily life applications: what are we missing? IEEE J. Biomed. Health Inform. 19, 6–21. 10.1109/JBHI.2014.2328317 25486653

[B35] Nieuwenhuis S. Forstmann B. U. Wagenmakers E.-J. (2011). Erroneous analyses of interactions in neuroscience: a problem of significance. Nature neuroscience 14:1105 10.1038/nn.2886 21878926

[B36] Ojeda A. Bigdely-Shamlo N. Makeig S. (2014). MoBILAB: an open source toolbox for analysis and visualization of mobile brain/body imaging data. Front. Hum. Neurosci. 8:121. 10.3389/fnhum.2014.00121 24634649 PMC3942646

[B37] Ojha S. Fainberg H. P. Sebert S. Budge H. Symonds M. E. (2015). Maternal health and eating habits: metabolic consequences and impact on child health. Trends Mol. Med. 21, 126–133. 10.1016/j.molmed.2014.12.005 25662028

[B38] Parada F. Rossi A. (2018). If neuroscience needs behavior, what does psychology need? Front Psychol. 9:433. 10.3389/fpsyg.2018.00433 29643829 PMC5883085

[B39] Pernet C. R. Chauveau N. Gaspar C. Rousselet G. A. (2011). LIMO EEG: a toolbox for hierarchical linear modeling of electroencephalo- graphic data. Comput. Intell. Neurosci. 2011, 10–11. 10.1155/2011/831409 PMC3049326 21403915

[B40] Pernet C. R. Wilcox R. Rousselet G. A. (2013). Robust correlation analyses: false positive and power validation using a new open source matlab toolbox. Front. Psychol. 3:606. 10.3389/fpsyg.2012.00606 23335907 PMC3541537

[B41] Pizzamiglio S. Naeem U. Abdalla H. Turner D. L. (2017). Neural correlates of single-and dual-task walking in the real world. Front. Hum. Neurosci. 11, 460. 10.3389/fnhum.2017.00460 28959199 PMC5603763

[B42] Satel S. Lilienfeld S. O. (2013). Brainwashed: The Seductive Appeal of Mindless Neuroscience. Basic Civitas Books.

[B43] Varela F. J. (1996). Neurophenomenology: a methodological remedy for the hard problem. J. Consciousn. Stud. 3, 330–349.

[B44] Volkow N. D. Swanson J. M. Evins A. E. DeLisi L. E. Meier M. H. Gonzalez R.. (2016). Effects of cannabis use on human behavior, including cognition, motivation, and psychosis: a review. JAMA Psychiatry 73, 292–297. 10.1001/jamapsychiatry.2015.3278 26842658

[B45] Wagenmakers E.-J. (2007). A practical solution to the pervasive prob- lems ofp values. Psychon. Bull. Rev. 14, 779–804. 10.3758/BF03194105 18087943

[B46] Walsh C. G. Ribeiro J. D. Franklin J. C. (2017). Predicting risk of sui- cide attempts over time through machine learning. Clin. Psychol. Sci. 5, 457–469. 10.1177/2167702617691560

[B47] Wilcox R. R. Rousselet G. A. (2018). A guide to robust statistical methods in neuroscience. Curr. Protocols Neurosci. 82, 8–42. 10.1002/cpns.41 29357109

[B48] Yarkoni T. (2009). Big correlations in little studies: inflated fmri correlations reflect low statistical power—commentary on Vul et al. (2009). Perspect. Psychol. Sci. 4, 294–298. 10.1111/j.1745-6924.2009.01127.x 26158966

[B49] Zuker C. S. (2015). Food for the brain. Cell 161, 9–11. 10.1016/j.cell.2015.03.016 25815979

